# Diagnostic value of piezogenic papules in childhood: A scoping review and analysis of existing data

**DOI:** 10.1186/s12887-026-07414-0

**Published:** 2026-08-12

**Authors:** Elke Schubert-Hjalmarsson, Ann-Charlott Söderpalm

**Affiliations:** 1 Department of Health and Rehabilitation, Division of Physiotherapy, Institute of Neuroscience and Physiology, University of Gothenburg, Gothenburg, Sweden; 2 Department of Physiotherapy, Paediatric Health Professions and Paediatric Radiology, Sahlgrenska University Hospital, Region Västra Götaland, Gothenburg, Sweden; 3 Department of Orthopedics, Institute for Clinical Sciences, Sahlgrenska Academy at University of Gothenburg, Gothenburg, Sweden

**Keywords:** Piezogenic papules, childhood, diagnostic markers, Scoping review, Heel papules, Pediatric diagnosis

## Abstract

**Introduction:**

Piezogenic papules (PP) are soft, skin-colored or yellowish herniations of subcutaneous fat appearing under pressure, commonly on the heels. While usually benign, they may cause pain and have been associated with Ehlers-Danlos syndrome. PP are included in the 2017 diagnostic criteria for hypermobile Ehlers-Danlos syndrome (hEDS), and in the 2023 framework for pediatric joint hypermobility, but their prevalence in healthy children and their clinical threshold remain unclear.

**Aim:**

To map current knowledge on the prevalence of PP in healthy children and adolescents and to analyze previously collected clinical data from healthy adolescents and those with Hypermobility Spectrum Disorder (HSD) or hEDS.

**Methods:**

A scoping review was conducted. Peer-reviewed studies reporting PP prevalence or clinical characteristics in healthy children and adolescents (0–17 years) or including healthy controls were included. Additionally, clinical data from adolescents assessed for hEDS (2017 criteria) and healthy controls were analyzed descriptively.

**Results:**

Of 707 identified articles, four observational studies and five case reports met the inclusion criteria. Across the reviewed studies, PP prevalence in children ranged from 16 to 72%, was generally asymptomatic. In the empirical cohort, PP were observed in 76% of individuals with HSD/hEDS and 60% in the control group, corresponding to a prevalence difference of 16% points (95% CI − 4 to 36; *p* = 0.163).

**Conclusion:**

PP are common and largely asymptomatic in children and adolescents. Although presumably more pronounced in individuals with HSD/hEDS, no statistically significant differences between groups were identified. Current evidence does not clearly support a diagnostic value, highlighting the need for further studies.

**Trial registration:**

Open Science Framework - OSF. 2025 May 12. osf.io/8zp25.

**Supplementary Information:**

The online version contains supplementary material available at 10.1186/s12887-026-07414-0.

## Introduction

Piezogenic papules (PP) are small, soft, skin-colored or yellowish protrusions that typically appear on weight-bearing areas, such as the heels. They are caused by herniation of subcutaneous fat through the dermis under pressure [1]. They present as firm, yellowish to skin-colored papules on the lateral, medial, or posterior heel, become more prominent with full weight-bearing, and resolve when pressure is relieved [2]. While commonly considered benign and asymptomatic, PP can, in some cases, cause discomfort or pain [3].

PP have been reported in individuals with connective tissue disorders, including Ehlers-Danlos syndrome (EDS) and Hypermobility Spectrum Disorder (HSD) [4, 5]. In the 2017 diagnostic criteria for hypermobile Ehlers-Danlos syndrome (hEDS), PP are listed among a group of skin features that support diagnosis [5]. However, the criteria do not define how prominent or symptomatic the papules must be to be clinically relevant, and no threshold for diagnostic significance has been established [6, 7].

The recently proposed pediatric joint hypermobility framework represents an updated approach for non-skeletally mature individuals, replacing earlier use of HSD/hEDS terminology in this age group. As the included data were collected prior to the introduction of the 2023 pediatric joint hypermobility framework, the terminology HSD/hEDS is retained throughout the manuscript to reflect the diagnostic criteria applied at the time of data collection. The new framework continues to include PP as one of six skin and tissue signs in children being evaluated for pediatric joint hypermobility, noting that three of six findings are needed to fulfill the skin and tissue criterion [7]. However, the authors emphasize a critical gap in normative data: the prevalence of PP in healthy children and adolescents remains unclear. Without reliable prevalence estimates, the specificity and diagnostic weight of PP remain uncertain.

Previous findings suggest a wide range in the frequency of PP observed among children. Van Straaten et al. [8] reported that up to 70% of healthy children in their cohort exhibited one or two visible PP. These observations underscore the need for better understanding of the clinical relevance and prevalence of PP in pediatric populations, particularly in relation to hEDS/HSD diagnostic evaluation.

### Aim

The aim of this study was to conduct a scoping review and to analyze previously collected clinical data from adolescents with HSD or hEDS. The study aimed to 1: systematically map the research done on the prevalence of PP in healthy children and adolescents, and 2: supplement this with empirical data in order to evaluate the occurrence of PP among both adolescents with HSD/hEDS and a healthy control group. The combined findings are intended to contribute to a more informed understanding of the prevalence of PP in pediatric populations.

## Methods

This study consisted of two parts: (1) a scoping review of the literature on PP in children and adolescents, and (2) an analysis of previously collected clinical data from adolescents assessed using the 2017 diagnostic criteria for hEDS. The study was prospectively registered with the Open Science Framework on May 12, 2025 (osf.io/8zp25).

### Scoping review

A scoping review methodology was chosen as it is appropriate for mapping the existing literature across study designs and for providing an overview of the available evidence. This approach allows identification of knowledge gaps and characterization of the evidence base without restricting inclusion based on study design or requiring synthesis of effect estimates.

The scoping review followed the methodological framework outlined by Arksey and O’Malley [9] and was reported in accordance with the PRISMA-ScR (Preferred Reporting Items for Systematic Reviews and Meta-Analyses extension for Scoping Reviews) checklist [10]. To ensure methodological transparency and replicability, the study protocol was registered with the Open Science Framework (OSF) [11].

Studies were considered eligible if they met the following inclusion criteria based on the PCC (Population–Concept–Context) framework: (a) original, peer-reviewed research; (b) the Population consisted of healthy children and adolescents aged 0–17 years; (c) the Concept comprised studies investigated the prevalence and/or clinical characteristics of PP of the heels; and (d) the Context included clinical and/or research settings, with no restrictions on publication date. The context further included studies published in, English, Swedish, Danish, Norwegian, or German. Studies were excluded if they focused exclusively on adult populations, were restricted to diagnosed patient groups, or were published in languages other than those specified above.

A systematic search strategy was developed in collaboration with a research librarian and applied across three databases: PubMed, EMBASE, and Scopus. Search terms included variations and combinations of keywords such as “piezogenic”, “papules”, “foot”, and “pedal” (Table 1) and the search was conducted in May 2025. Due to differences in indexing systems across databases, age-related concepts were identified using both controlled vocabulary (where available, e.g., PubMed and EMBASE) and free-text terms. As Scopus does not use a controlled vocabulary system, age-related concepts were searched using free-text terms only. Database-specific limits were applied where appropriate; for example, the document type filter “article” was used in EMBASE and Scopus to exclude conference abstracts and other non-article records. Despite these adaptations, the same core search concepts and eligibility criteria were applied consistently across all databases.

**Table 1:**
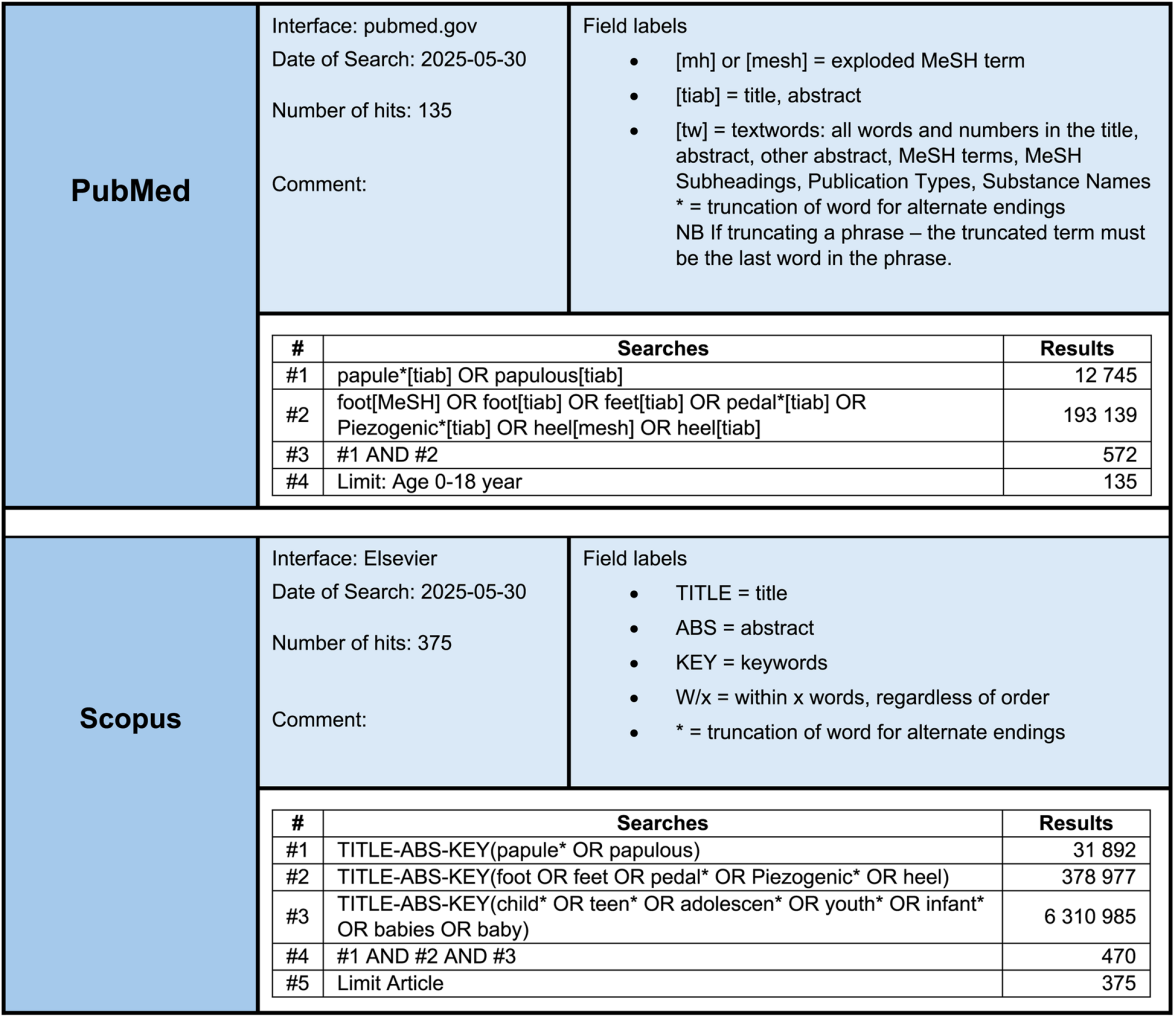

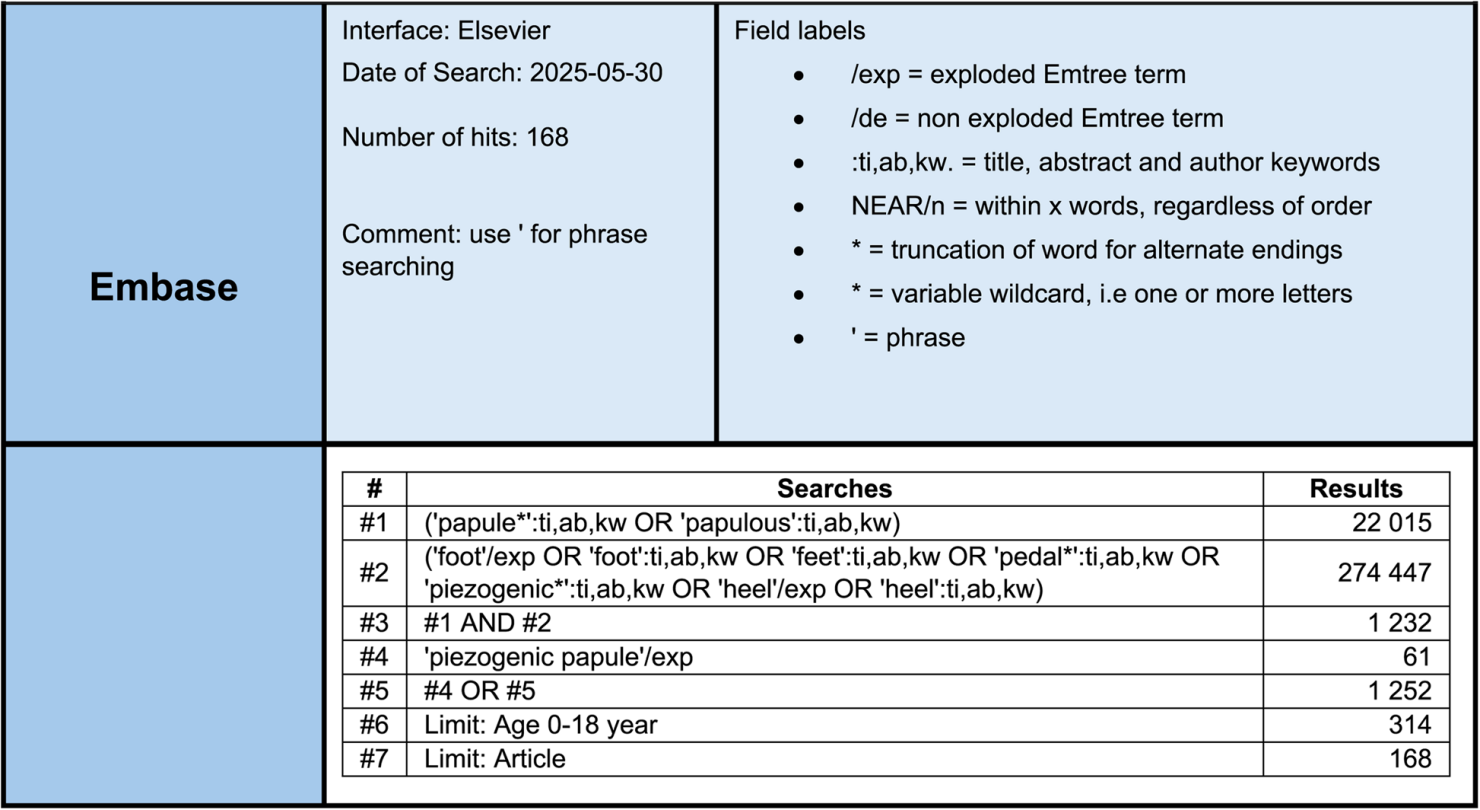
Search strategy across databases and results from the initial search

To ensure that newly published studies were captured, an updated search using the same search strategy and databases was conducted for the period June 2025 to January 2026. The search results were screened and agreed upon independently by two reviewers, first by title and abstract, and subsequently at the full-text level. Reference lists of the included studies were systematically checked for additional relevant articles. Authors were contacted, if necessary, to clarify pediatric-specific data.

Data from the included studies were extracted using a standardized data extraction form that captured information such as author, study design, year, country, sample characteristics, definition and reported prevalence of PP, the methods used for identification and assessment. The extracted data were synthesized and presented descriptively in both narrative and tabular form.

### Empirical data analysis

In parallel with the scoping review, we analyzed previously collected clinical data from a cohort of adolescents who had been assessed for hEDS using the 2017 diagnostic criteria. The data originated from a prior published study, in which physical examinations were conducted on adolescents diagnosed with HSD or hEDS and age-matched healthy controls during the same assessment sessions [12]. While the original publication did not report on PP, this feature had been systematically documented at the time of examination. PP was assessed under full weight-bearing in a standing position and graded according to a 4-point scale: 0 = no PP, 1 = mild PP, 2 = moderate PP, and 3 = severe PP (Appendix 1 for image examples).

### Statistical methods for empirical data analysis

Descriptive statistics were used to summarize the occurrence and distribution of PP. Descriptive data are reported as median and interquartile range, or as frequency and percentage (%). Between-group comparisons for ordinal data were performed using the Mann–Whitney U test, while categorical variables were analysed using Fisher’s exact test. Statistical analyses were conducted using IBM SPSS Statistics version 28 (IBM SPSS Data Collection). These results were integrated into the discussion to contextualize the findings from the scoping review.

## Results

**Fig. 1:**
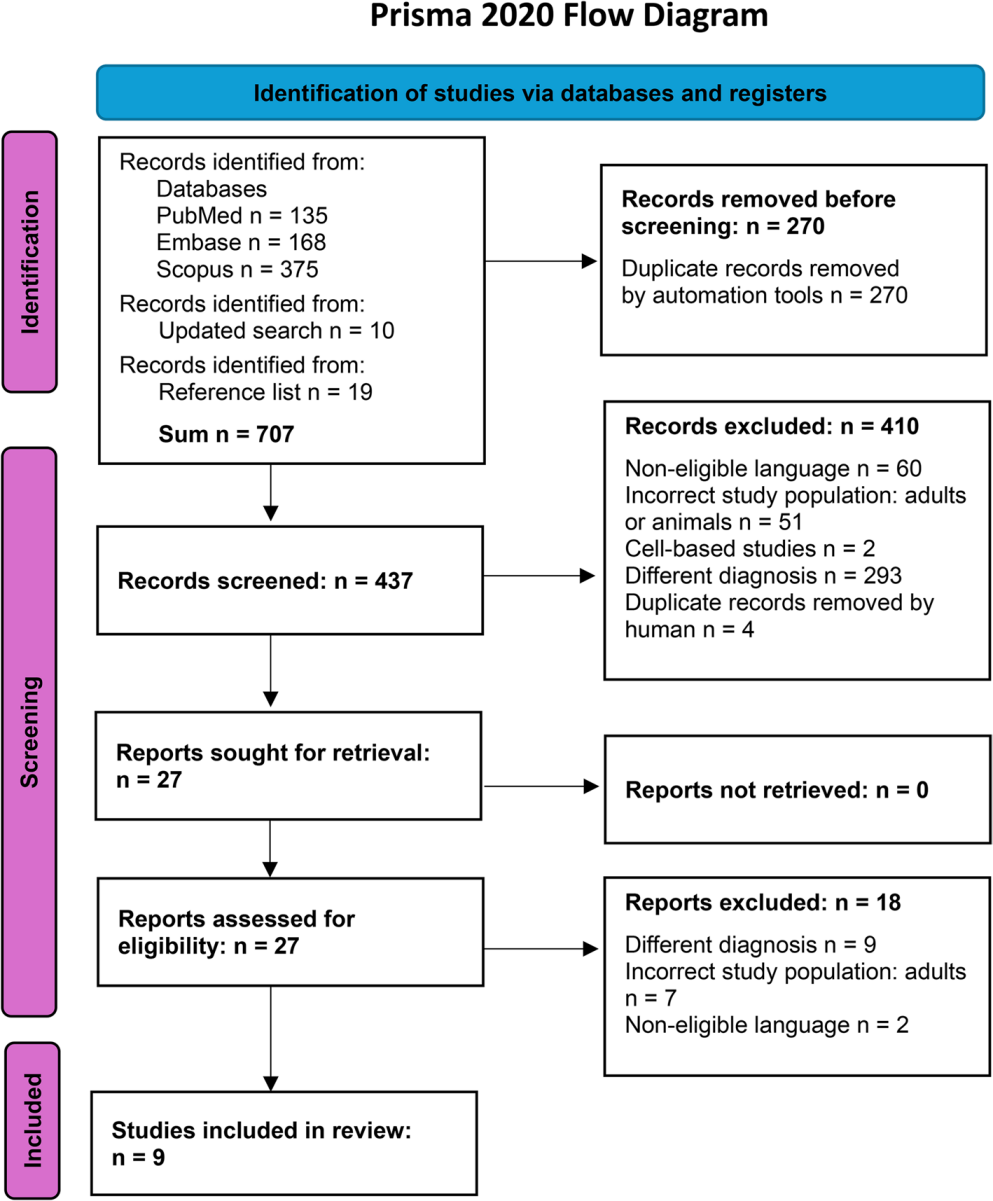
PRISMA flow diagram illustrating the study selection process for the scoping review

The search identified 707 articles. The initial search yielded 678 articles, of which 270 duplicates were removed. An additional 10 articles were identified through the complementary search, and a further 19 through screening of reference lists. The titles and abstracts of a total of 437 articles, identified across all three search strategies, were screened for eligibility based on the inclusion and exclusion criteria. Based on this screening, 410 articles were excluded. The full texts of the remaining 27 articles were assessed for eligibility, and 9 were included in this scoping review as presented in the flow diagram (Fig. 1). Excluded full-text articles are reported in Appendix 2.

### Characteristics of included studies

Nine observational studies reported data relevant to the prevalence and characteristics of PP of the heels in children (Table 2). Four of these nine studies examined larger populations. Of these, two studies exclusively examined pediatric populations [8, 13], whereas the other two included a mixed-age population and did not stratify results by age [14, 15]. The authors of the mixed-age studies were successfully contacted; however, pediatric-specific data could not be obtained, as the authors reported that these data were not available. Despite this limitation, the latter studies were included due to their relevance to the overall occurrence of PP. Five studies were single-case or small case series reports [16–20]. Three of the case reports exclusively examined young children [17–19], while the other two also included adults [16, 20]. Although some case reports included only one or a few participants, they provide detailed clinical descriptions relevant to the presentation, size, and symptomatology of PP.

**Table 2:** Characteristics of included studies

Author Typ of study	year	country	Population/ settings	definition of PP	reported prevalence of PP	methods used for assessment	Conclusion
Brzezinski [13] Cross-sectional study	2013	Poland	50 children age 1–10 (parents *n* = 68 age 24–46) – parent’s members of a sport club	Piezogenic pedal Nodules or Papules – flesh-coloured or yellowish, localized along the sole of the feet	8 of 50 (16%) age 4–10 had PP. 75% girls, 25% boys; non painful; 2 of 8 had past foot injuries; 2 of 8 had relatives with connective tissue diseases	Not described	PP could be related to intensiveness of physical activity
Van Straaten [8] Cross-sectional study	1991	Nether lands	322 Children age 4–13 – primary school	“Pressure-induced papules that appear when heels bear weight”	232 of 322 (72%) had one or more PP (68% of girls, 76% of boys) Average number of PP 5, mean PP diameter 3.3 mm, range 1–10 mm. – non painful 14 children were hypermobile (4.3%) 10 of these children has PP – same ratio for children with and without hypermobility	Each child was visually screened for PP while standing on a hard wooden bench. Number, size and location of PP were recorded. Noted if PP painful.	PP are presented in the majority of healthy children. It could not be confirmed that connective tissue fragility increase number och size of PP. No difference in number and prevalence of PP in hypermobile and non-hypermobile children
Cho [16] case series	2009	Korea	4 3 children age 3 female, 4 female, 7 male (1 adult) without signs of collagen tissue defect	skin-coloured papules	3 children with several, discreet, pain free papules, diameter 2–3 mm	Participants stand or direct pressure on the heel, ultrasonography assessment	Plantar PP in children may represent a normal developmental variant due to immature dermal–subcutaneous integrity; long-term follow-up is needed.
Gibney [17] case rapport	1996	USA, Missouri	1 Age 17 male	skin-coloured papules	Ca. 20 PP Diameter 3–6 mm Medial-lateral of the heel Rare episodes of minimal pain in the heels	Noted standing	
Wolf [18] case rapport	1986	Yugoslavian	1 Age 13 female	light-coloured, soft papules	Ca. 6 PP, bilateral, pea-sized painful	Noted standing	
Stintz [19] case rapport	1975	Germany	1 Age 12 male	Light-coloured papules	Multiple, more or less prominent, light-colored, lens- to pea-sized, hemispherical, firm papules. More pronounced after prolonged weight-bearing.	Noted under pressure, (i.e., when standing)	
Cohen [20] case rapport	1970	USA NY	4 1 child Age 13 Female (3 adults)	Soft skin-coloured papules	Several soft, skin-coloured papules on the sides of the feet	Standing, Disappeared when sitting No pain	It is possible that pain is an unusual feature of a condition that may be relatively common.
Zaidi* [14] Cross-sectional study	1993	Pakistan	100 Mixed-age population (age range 11–60 years) Outpatients without history of collagen tissue defect nor exposed to any vigorous physical activity	PP present as soft skin-coloured papules which appear on application of pressure and disappear on removal of force	80 of 100 (80%) had pedal PP 86 at the wrist, 74 of them had both pedal and wrist PP non painful	Participants stand and apply pressure on the heels	Painless PP are a common finding. Only a few of these papules become painful.
Plewig* [15] Cross-sectional study	1972	Germany	> 100 Mixed-age population (age range 2–86 years) “Skin-healthy” subjects, out- and inpatients	Hemispherical protruding papules during weight-bearing; skin-coloured to porcelain white.	Present in approximately 20% across all age groups; occurs in both females and males of all ages, but less frequently in children. Characterised by 1–20 hemispherical, raised papules. Non pinfull	Inspection of the medial, dorsal, and lateral heel margins before and during weight-bearing (toe-standing, heel-standing)	PP are common across all age groups and does not cause subjective complaints.

* This study included a mixed-age population. Although the authors were contacted, age-specific data for children were no longer available. Results from this study should be interpreted with caution

*Abbreviation PP*  Piezogenic papules

### Definition and identification of piezogenic papules

Across studies, PP were defined as pressure-induced, skin-colored or yellowish papules located on the medial and lateral aspect of the heel and appearing during weight-bearing. No included studies reported specific diagnostic thresholds for the number or size of papules required for inclusion. Instead, identification was consistently based on the visual presence of PP during weight-bearing or manual pressure. Among the case reports, Cho et al. [16] also included ultrasonography assessment. Four studies documented the number, size, and pain status of PP [8, 16–18].

### Prevalence and clinical characteristics in children

Among the two cross sectional studies focusing exclusively on children, reported prevalence of PPs across participant samples ranged from 16% to 72% (Table 3) [8, 13]. In larger pediatric populations, PP were generally small and asymptomatic, with no consistent differences related to hypermobility or sex, although some studies reported a slightly higher frequency in girls. The five case reports provide additional clinical observations: in young children, papules were reported as discrete, small, and painless [16, 19, 20], while in older children and adolescents, papules were sometimes larger, more numerous, or associated with occasional pain [17, 18]. These observations highlight the variability of PP presentation in children; the available data indicate that PP are common and that their visibility, size, and number are primarily influenced by mechanical factors rather than age or sex.

**Table 3:** Summary of participants, demographics, and outcomes in included studies

	Type of study	Number of participants	age	sex	Piezogenic Papules	Painful
Paediatric population						
Brzezinski [13]	Cross-sectional study	50	1–10	Not reported	8 (16%) 6 (75%) female 2 (25%) male	non
Van Straaten [8]	Cross-sectional study	322	4–13	136 female 186 male	232 (72%) 92 (68%) female 140 (76%) male	non
Cho [16]	Case series	3	3–7	2 female 1 male	3 (100%)	non
Gibney [17]	Case report	1	17	Male	1 (100%)	non
Wolf [18]	Case report	1	13	Female	1 (100%)	painful
Stintz [19]	Case report	1	12	Male	1 (100%)	painful
Cohen [20]	Case report	1	13	Female	1 (100%)	non
Total		379			251	2 (< 1%)
Mixed-age population						
Ziadi* [14]	Cross-sectional study	100	11–60	Not reported	80 (80%) Age and sex not reported	non
Plewig* [15]	Cross-sectional study	> 100	2–86	Not reported	> 20 (20%) Age and sex not reported	non
Total		200			100 (50%)	0

* This study included a mixed-age population. Although the authors were contacted, age-specific data for children were no longer available. Results from this study should be interpreted with caution

### Empirical Data

In total, 84 adolescents aged 14–17 years were assessed for the presence of PP, of whom 37 (44%) were in the HSD/hEDS group and 47 (56%) were in the healthy control group; group characteristics and between-group differences are presented in Table 4.

**Table 4:** Characteristics of the empirical study population and prevalence of Piezogenic Papules

		HSD/hEDS group (*n* = 37)	Control group (*n* = 47)	Test statistic ^a^	*P* value ^a^
		Median (IQR)	Median (IQR)		
Age (years)		16.4 (14.8–17.0)	15.3 (14.0-16.2)	615.5	**0.022**
Beighton score (0–9)		6 (4–7)	2 (1–4)	314.0	**< 0.001**
Beighton score male		5 (2–6) *n* = 10	1 (0–3) *n* = 13	18.5	**0.003**
Beighton score female		6 (4–7) *n* = 27	2 (2–4) *n* = 34	164.5	**< 0.001**
Length (m)		1.7 (1.6–1.7)	1.7 (1.6–1.8)	701.5	0.130
Weight (kg)		58.5 (50.8–72.9)	59 (52.1–67.5)	854.0	0.889
BMI (kg/m²)		21.2 (18.5–25.6)	20 (18.9–23.4)	732.5	0.217

		Frequency (%)	Frequency (%)		
Sex	male	10 (27)	13 (28)	864.0	0.949
	female	27 (73)	34 (72)		

Piezogenic papules		Frequency (%)	Frequency (%)	Difference % (95% CI)	P value ^b^
None		9 (24)	19 (40)		
Mild		15 (41)	22 (47)		
Moderate		12 (32)	6 (13)		
Severe		1 (3)	0		
Presence of PP (total)		28 (76)	28 (60)	16 (-4 to 36)	0.163

*Abbreviation*: HSD = Hypermobility spectrum disorder; hEDS = hypermobile Ehlers-Danlos syndrome; BMI = Body Mass Index; IQR = Inter quartile range; PP = Piezogenic papules; OR = Odds ratio; CI = Confidence interval

^a^ Mann Whitney U test statistic for group differences; ^b^ Fisher’s exact test; Bold values indicate statistically significant results (*p* < 0.05)

The results from the empirical data showed that 28 participants (76%) in the HSD/hEDS group had PP, of which 15 (41%) were classified as mild, (32%) as moderate, and 1 (3%) as severe. In the healthy control group, 28 participants (60%) presented with PP, of which 22 (47%) were classified as mild and 6 (13%) as moderate. None of the PP were associated with pain in either group (Table 3). No statistically significant difference in PP prevalence was observed between the HSD/hEDS and control groups (76% vs. 60%; prevalence difference 16% points, 95% CI − 4 to 36; *p* = 0.16).

## Discussion

Few studies have investigated the prevalence of PP in children. Available literature suggests that PP are common in pediatric populations, with reported prevalence rates around 16–74% of the study population in healthy children. Van Stratten et al. [8] also referred to one additional study published in French that was excluded from the inclusion criteria for this review; in that study, according to Van Stratten et al. examined Delalande 100 healthy children of walking age and identified PP in 79.5% of girls and 83% of boys [21]. Similarly, a review by Gibney et al. summaries that the prevalence of PP is approximately 70% in children and 60% in adults, with only a small proportion of cases being painful [17].

In contrast to the findings of Van Straaten et al., who reported no association between hypermobility and the prevalence, number, or size of PP [8], the empirical data demonstrated that although the overall prevalence of PP did not differ between groups, adolescents with HSD/hEDS exhibited a higher frequency of more pronounced or severe forms of PP compared with controls. Importantly, no painful PP were observed in either group, indicating that pain may not be a common feature of PP in adolescents.

Although the present scoping review focuses on children and adolescents, the literature also indicates a high prevalence of asymptomatic PP in adults. Studies by Dockery and Diana [22] and by Schlappner et al. [3] demonstrate that PP are common in adult populations and are frequently painless, further supporting the notion that PP are often an incidental finding across age groups.

Some studies have explored the diagnostic value of PP in individuals with EDS, where images typically show more extensive, larger, and occasionally painful papules [23, 24]. Given their high prevalence and frequent lack of symptoms, it may be important to more clearly define when PP should be considered a diagnostically meaningful finding, for example when they are moderate to severe and/or painful.

Kahana et al. reported an increased prevalence of painful PP in a cohort of 29 adults with EDS [24]. In contrast, the empirical data included in the present review did not demonstrate painful PP in children with HSD/hEDS; however, the papules were in some cases described as more pronounced. It has been hypothesized that painful PP may be larger than asymptomatic papules and may arise from the fusion of smaller fat chambers [17]. Further research is needed to determine whether children with PP and HSD/EDS are at increased risk of developing painful PP later in life. At the same time, it is important to consider in clinical practice that heel pain is relatively common in childhood [25, 26], meaning that PP may coexist with other, more established causes of pain.

### Strengths and limitations

To ensure a high-quality and systematic search process, an experienced university librarian was consulted during the development of the search strategy and conducted the literature searches across three databases. This focused selection ensured manageability and consistency but may also have limited the breadth of retrieved studies. The choice of databases presents both strengths—such as targeting relevant health-related literature—and weaknesses, as some potentially relevant studies may be indexed elsewhere.

No restrictions were applied regarding publication year, allowing the inclusion of both recent and older studies indexed in the selected databases. However, the search was limited to certain databases, and only articles published in selected languages were included due to time constraints and translation costs. Consequently, some relevant studies published in other languages may have been missed.

As a scoping review, this study does not aim to synthesize evidence or assess the methodological quality of included studies. Consequently, it cannot determine whether the findings are robust or generalizable. Further research is needed to evaluate the diagnostic value of PP in children and adolescents.

## Conclusion

In conclusion, based on the available data, PP appear to be a common and mostly asymptomatic finding in children and adolescents. Although more pronounced or extensive PP may be observed in individuals with HSD/hEDS, no statistically significant differences between groups were identified, and the clinical significance therefore remains uncertain. Furthermore, the available literature does not clearly distinguish between pain directly attributable to PP and more general musculoskeletal or heel pain, limiting interpretation of reported symptoms. Their limited discriminatory value calls into question the usefulness of PP as a diagnostic marker in pediatric populations. Future research should address the prevalence and severity of PP, clarify the nature of associated pain, and evaluate their potential role within current pediatric diagnostic frameworks.

## Supplementary Information

Supplementary Material 1.

Supplementary Material 2.

## Data Availability

The datasets used and analysed during the current study are available from the corresponding author, Elke Schuber-Hjalmarsson, on reasonable request.

## Abbreviations

EDS: Ehlers-Danlos syndrome
hEDS: Hypermobile Ehlers-Danlos syndrome
HSD: Hypermobility spectrum disorder
PP: Piezogenic papules
