# Supplementary material for: Diagnostic value of piezogenic papules in childhood: A scoping review and analysis of existing data
Source: BMC Pediatr. 2026 Aug 12;26:734. doi: 10.1186/s12887-026-07414-0 (PMC13465072; doi:10.1186/s12887-026-07414-0)
Supplement: Supplementary file 1 [file 12887_2026_7414_MOESM1_ESM.docx]

**Appendix 1: Clinical Examples and Grading of Piezogenic Papules**

**Mild:**

*single or few visible piezogenic papules*

1:

**Moderate:**

*several piezogenic papules with clear visibility*

2:

**Severe:**

*multiple prominent, large piezogenic papules*

3:
