# Supplementary material for: Diagnostic value of piezogenic papules in childhood: A scoping review and analysis of existing data
Source: BMC Pediatr. 2026 Aug 12;26:734. doi: 10.1186/s12887-026-07414-0 (PMC13465072; doi:10.1186/s12887-026-07414-0)
Supplement: Supplementary file 2 [file 12887_2026_7414_MOESM2_ESM.docx]

**Appendix 2**: List of excluded full-text articles

| Author | Year | Title | Abstract available | Reason for exclusion |
| --- | --- | --- | --- | --- |
| Alowirdi, F | 2020 | Asymptomatic plantar nodules in a 1-year-old healthy girl | No | Different diagnosis / condition |
| Conde-Taboada, A | 2007 | Hypertrophic infantile pedal papules | No | Different diagnosis / condition |
| Greenberg, S | 2005 | Infantile pedal papules | Yes | Different diagnosis / condition |
| Klaber, M. R | 1984 | Some spots of no significance | Yes | Different diagnosis / condition |
| Kreher, M. A | 2023 | Regressing Papules on the Sole of the Foot: Challenge | No | Different diagnosis / condition |
| Mendes, A. R | 2023 | Small white bumps on the heels of an adolescent | No | Different diagnosis / condition |
| Nyers, E. S | 2020 | Asymptomatic pink heel papules and nodules in a newborn infant | No | Different diagnosis / condition |
| Singh, SK | 2002 | Painless piezogenic pedal papules in a patient with rheumatic heart disease | No | Different diagnosis / condition |
| Yen, E | 2011 | A male infant with bumps on his feet | Yes | Different diagnosis / condition |
|  | | | | |
| Dockery, G. L | 1978 | Painful piezogenic papules | No | Incorrect study population: adults |
| Grant DJ | 1970 | Piezogenic pedal papules | No | Incorrect study population: adults |
| Lebovits PE | 1982 | Piezogenic pedal papules | No | Incorrect study population: adults |
| Pontious J | 1990 | Piezogenic pedal papules extending into the arch. Case report and discussion | No | Incorrect study population: adults |
| Schubert E | 1973 | Multiple Fettgewebshernien der Ferse. (Sogenannte "painful piezogenic pedal papules" [Multiple adipose tissue hernias of the heel. (So-called "painful piezogenic pedal papules")] | No | Incorrect study population: adults |
| Shelley WB | 1968 | Painful feet due to herniation of fat | No | Incorrect study population: adults |
| Stern J | 1977 | Painful piezogenic pedal papules. A case history | No | Incorrect study population: adults |
|  | | | | |
| Dreno B | 1984 | Painful piezogenic pedal papules [Painful piezogenic pedal papules] | No | Language not eligible |
| Vittori, F | 1985 | [Piezogenic papules of the feet] | No | Language not eligible |
